# Supplementary figures and images for: Pharmacological management of acute spinal cord injury: a longitudinal multi-cohort observational study
Source: Sci Rep. 2023 Apr 3;13:5434. doi: 10.1038/s41598-023-31773-8 (PMC10070428; doi:10.1038/s41598-023-31773-8)

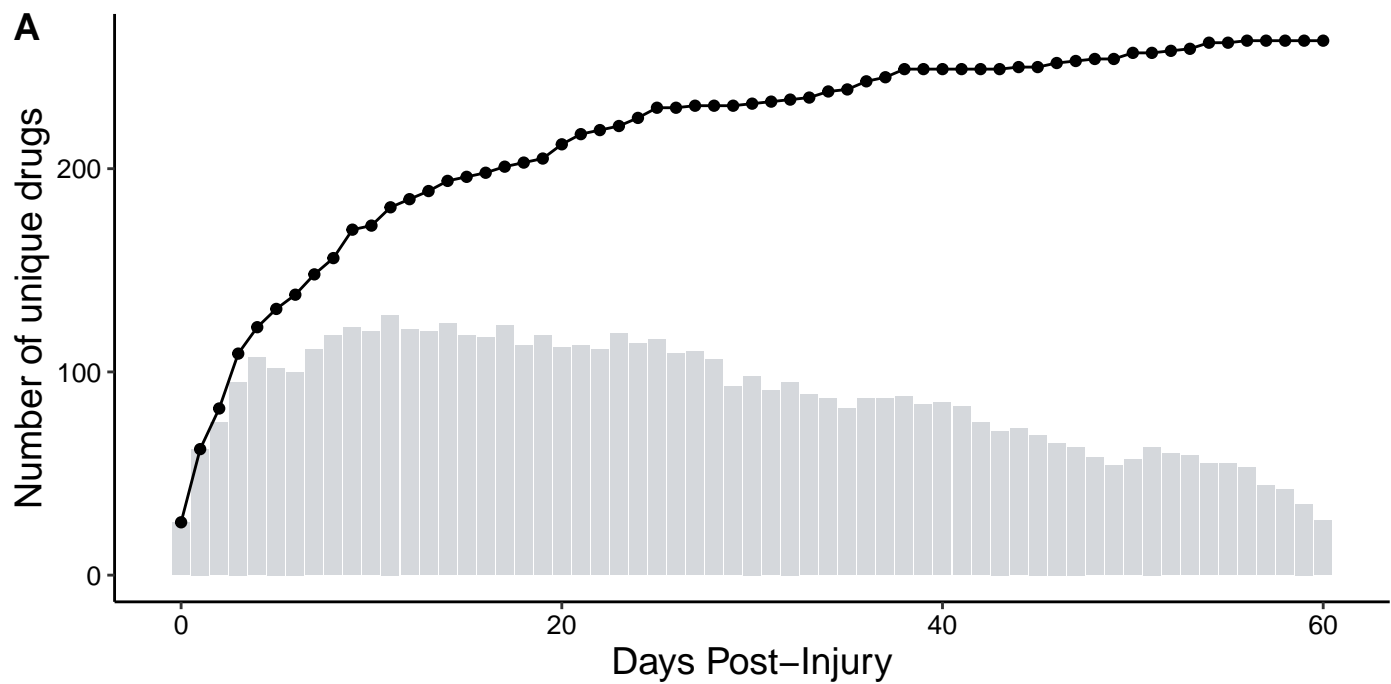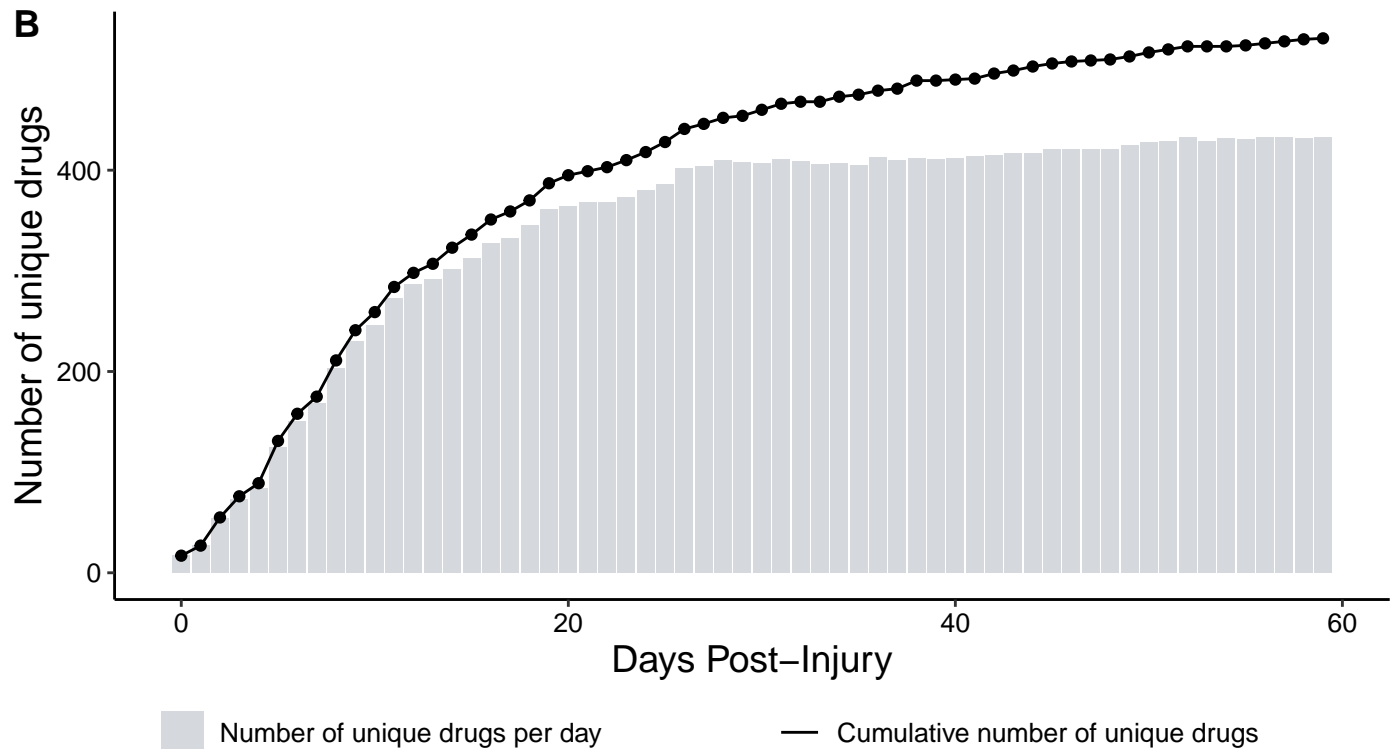

Supplement: Supplementary file 1 — Supplementary Figure 1. [file 41598_2023_31773_MOESM1_ESM.pdf]
