# Supplementary material for: Pharmacological management of acute spinal cord injury: a longitudinal multi-cohort observational study
Source: Sci Rep. 2023 Apr 3;13:5434. doi: 10.1038/s41598-023-31773-8 (PMC10070428; doi:10.1038/s41598-023-31773-8)
Supplement: Supplementary file 10 — Supplementary Legends. [file 41598_2023_31773_MOESM10_ESM.docx]

**Supplementary Figure 1. Number of unique drugs per day and cumulative number of unique drugs for (A) the Sygen trial and (B) SCIRehab Study.**
